# Soluble carbohydrate content variation in *Sanionia uncinata* and *Polytrichastrum alpinum*, two Antarctic mosses with contrasting desiccation capacities

**DOI:** 10.1186/s40659-015-0058-z

**Published:** 2016-01-28

**Authors:** Paz Zúñiga-González, Gustavo E. Zúñiga, Marisol Pizarro, Angélica Casanova-Katny

**Affiliations:** Laboratorio de Micología y Micorrizas, Facultad de Ciencias Naturales y Oceanográficas and Laboratorio de Investigación en Agentes Antibacterianos, Facultad de Ciencias Biológicas, Universidad de Concepción, Barrio Universitario s/n, Concepción, Chile; Departamento de Biología, Facultad de Química y Biología, Universidad de Santiago, Alameda, 3363 Santiago, Chile; Núcleo de Estudios Ambientales, Universidad Católica de Temuco, Casilla 15-D, Temuco, Chile; Facultad de Química y Biología, Universidad de Santiago, Alameda, 3363 Santiago, Chile

**Keywords:** Antarctica, Antarctic vegetation, Bryophytes, Sugars

## Abstract

**Background:**

Cryptogamic vegetation dominates the ice-free areas along the Antarctic Peninsula. The two mosses *Sanionia uncinata* and *Polytrichastrum alpinum* inhabit soils with contrasting water availability. *Sanionia uncinata* grows in soil with continuous water supply, while *P. alpinum* grows in sandy, non-flooded soils. Desiccation and rehydration experiments were carried out to test for differences in the rate of water loss and uptake, with non-structural carbohydrates analysed to test their role in these processes.

**Results:**

Individual plants of *S. uncinata* lost water 60 % faster than *P. alpinum*; however, clumps of *S. uncinata* took longer to dry than those of *P. alpinum* (11 vs. 5 h, respectively). In contrast, rehydration took less than 10 min for both mosses. Total non-structural carbohydrate content was higher in *P. alpinum* than in *S. uncinata*, but sugar levels changed more in *P. alpinum* during desiccation and rehydration (60–50 %) when compared to *S. uncinata*. We report the presence of galactinol (a precursor of the raffinose family) for the first time in *P. alpinum.* Galactinol was present at higher amounts than all other non-structural sugars.

**Conclusions:**

Individual plants of *S. uncinata* were not able to retain water for long periods but by growing and forming carpets, this species can retain water the longest. In contrast individual *P. alpinum* plants required more time to lose water than *S. uncinata*, but as moss cushions they suffered desiccation faster than the later. On the other hand, both species rehydrated very quickly. We found that when both mosses lost 50 % of their water, carbohydrates content remained stable and the plants did not accumulate non-structural carbohydrates during the desiccation prosses as usually occurs in vascular plants. The raffinose family oligosaccarides decreased during desiccation, and increased during rehydration, suggesting they function as osmoprotectors.

**Electronic supplementary material:**

The online version of this article (doi:10.1186/s40659-015-0058-z) contains supplementary material, which is available to authorized users.

## Background

Over the last decades, Antarctica has become a natural laboratory for studying plant tolerance mechanisms under extreme conditions and climate change. In the Antarctic, the development of most life forms is limited due to abiotic factors such as low temperatures, frequent cycles of freezing and thawing, high radiation, strong winds, and extreme dryness; a dryness due in part to the lack of organic soil capable of water retention, in addition to the physiological drought caused by freezing [1]. All these elements contribute to low water availability for plant growth and cellular activities which represents one of the principal limiting factors for distribution of terrestrial vegetation [2].

The Antarctic flora is poor in vascular plants, with lichens, mosses, and liverworts dominating the landscape. Plant-lichen communities are distributed at ice-free sites along the west part of the Antarctic Peninsula and on the offshore islands of the maritime Antarctic [3]. Only a few lichen and moss species are capable of surviving the freezing temperatures and strong desiccation found further south [4].

King George Island forms part of the South Shetlands Archipelago in the maritime Antarctic, and is characterized by a semidesert landscape [5]. This island hosts 61 reported moss species located at sites that are humid, protected, and covered by relatively stable and partially organic soil [6]. *Sanionia uncinata* (Hedw.) Loeske and *Polytrichastrum alpinum* (Hedw.) G. L. Smith are frequently found on Fildes Peninsula. In predominantly bryophytic communities, *S. uncinata* grows on the borders of waterlogged areas as well as close to small water bodies, stream banks, and spots subject to melting-water runoff. *P. alpinum* grows preferentially on humid and rocky substrates and close to moraine peaks of glaciers or at dry sites [7, 8], but not in water-saturated soils. This species, together with the two native vascular plants *Deschampsia antarctica* Desv. and *Colobanthus quitensis* (Kunth.) Bartl., form the so-called herbaceous antarctic tundra [7]. In this context, *D. antarctica*, the antarctic hairgrass, is positively associated with moss beds along the Antarctic Peninsula which, have been shown to facilitate growth of *D. antarctica* seedlings in transplant experiments on Fildes Peninsula [9].

Bryophytes are characterized by a dominant gametophytic phase during their life cycle and a poorly developed vascular system. These plants are capable of easily losing and reabsorbing water through the cellular membrane. Mosses as poikilohydric organisms can rapidily adjust cellular water content in relation to air and environmental humidity [10, 11]. Their inability to maintain stable tissue water levels requires mosses to develop desiccation tolerance mechanisms, such as the total suspension of metabolic activity in order to survive water shortage [12]. Desiccation tolerance is more common in mosses than in homohydric plants (tracheophytes) [13]. The diurnal, monthly and seasonal periods of desiccation to which mosses are exposed determines their establishment and survival, especially in extreme environments such as the Antarctica [10, 13, 14]. According to Bewley [15], the following three properties of the protoplasm in cells are essential for desiccation tolerance: (1) keeping damage to a minimum during desiccation and rehydration, (2) maintaining cellular integrity during desiccation, and (3) activating repair mechanisms following rehydration. All mechanisms are ultimately focused on cellular protection and repair.

Among the mechanisms for cellular protection, soluble carbohydrate accumulation has been related to higher desiccation tolerance in plants [10, 16–18], seeds [16], angiosperm pollen [19], the gametophytes of certain mosses [11, 20, 21] and moss spores [22]. One of the reasons for this accumulation is that soluble carbohydrates contribute to cytoplasm vitrification [23], which facilitates the preservation of macromolecules and the maintenance of membrane integrity for prolonged periods [10, 11, 24, 25].

The role of sugars in the dehydration processes of higher plants has been extensively described [18]. Plants resistant to water loss accumulate soluble sugars that diminish the osmotic potential of the cell, hydrating macromolecules during desiccation stress [18]. However, mosses are poorly investigated in terms of the role of sugars in the processes of daily or seasonal dehydration and rehydration. As dominant species in many tundra communities on the ice-free soils of the maritime Antarctic, both *S. uncinata* and *P. alpinum* play fundamental ecological roles by changing soil properties [26], so understanding the functioning of these key species may also allow deeper insight into plant–plant interactions and the responses of the whole community to changes in water regime.

The present study investigated and compared the rate of water loss and uptake for *S. uncinata* which forms carpets at the wettest sites, and *P. alpinum* which grows on drier, sandy soil, forming small cushions, followed by measurements of changes in non-structural carbohydrate content and composition in both species in response to short term desiccation and rehydration. The results should not only contribute to predicting responses of the polar tundra ecosystem as a whole to climate change, but also reveal potential interactions between bryophytes and antarctic vascular plants as well as other groups of organisms such as springtails and mites.

## Results

### Desiccation and rehydration curves

**Fig. 1:**
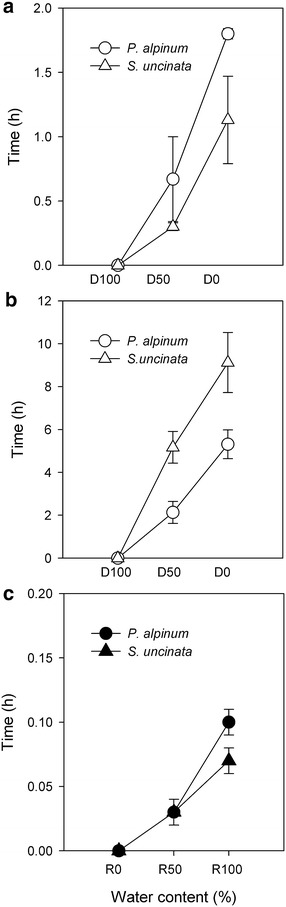
Time curves (h) for water loss and uptake in Antarctic mosses. Time required to reach every water level during desiccation (from D100 to D0) of **a** individual plants or **b** disc samples and **c** rehydration (R0–R100) of *Sanionia uncinata* and *Polytrichastrum alpinum*. D100, D50, D0, R0, R50 and R100 indicated the percentage of tissue water content. Values are means (n = 4 for disc samples and n = 6 for individual plant) ± SD

During the first desiccation experiment which compared individual plants, *S. uncinata,* an ectohydric moss took significantly less time to completely dehydrate than the endohydric *P. alpinum* [Fig. 1a; full desiccation (D0) reached after 1.13 ± 0.34 vs. 1.8 ± 0.04 h; put stats here (F_(1,66)_ = 63.55, p < 0.0001)]. However, desiccation took much longer when discs of both mosses were used (Fig. 1b; F_(1,66)_ = 193.2, p < 0.0001). In this case, clumps of *S. uncinata* reached D0 after eleven hours while *P. alpinum* took 5 h to desiccate (Fig. 1b). During rehydration both species needed only a few minutes (<6 min) to reach the highest water tissue content (R100) (Fig. 1c). The differences observed between species (F_(1,66)_ = 63.55, p < 0.0001), the type of samples (discs or individual plants) (F_(1,66)_ = 193.2, p < 0.0001) and which treatments (F_(5,66)_ = 332.7, p < 0.0001) were statistically significant. The interaction between the variables, that is, species by which samples (F_(1,66)_ = 95.5, p < 0.0001), species by treatments (F_(5,66)_ = 40.75, p < 0.0001), and samples by treatment (F_(2,66)_ = 245.47, p < 0.0001), were also significant. Moreover, the interactions between the three variables were as well significant.

### Carbohydrate content in mosses

**Fig. 2:**
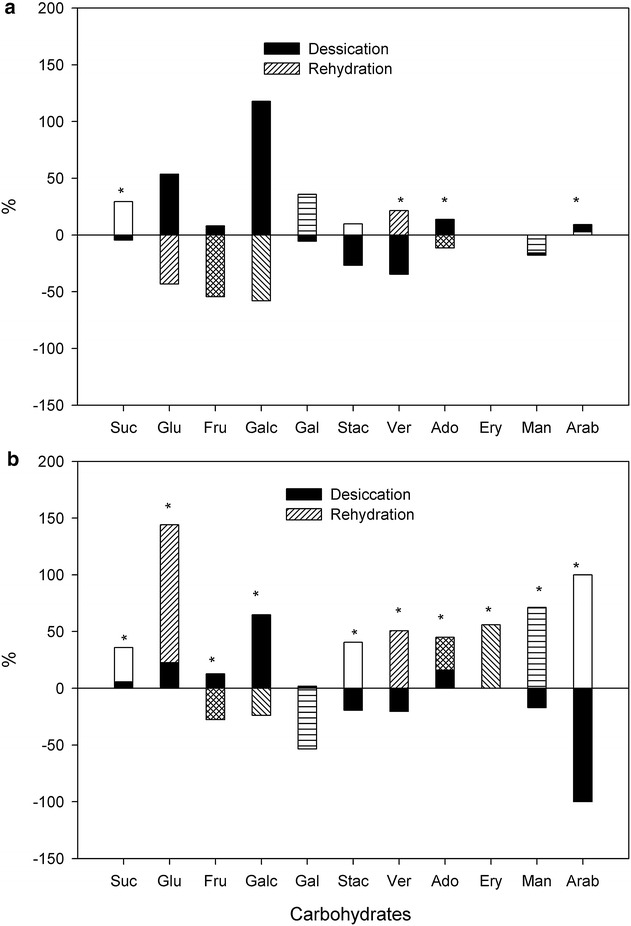
Percentage of change of each soluble carbohydrate in Antarctic mosses. In **a** *S. uncinata* and **b** *P. alpinum* between the start and the end of desiccation (D100–D0) and rehydration (R0–R100). Values are percentage according to carbohydrate content showed in Additional file 1: Table S1. *Indicate significant differences in Additional file 1: Table S1. *Su* sucrose, *Glu* glucose, *Fru* fructose, *Galc* galactose, *Gal* galactinol, *Ver* verbascose, *Stac* stachyose, *Ado* adonitol, *Eri* erithritol, *Man* mannitol, *Arab* arabitol

During dehydration and rehydration assays, non-structural carbohydrate content (NSC) differed significantly with treatment (F_(4,30)_ = 42.5; p < 0.0001) and between species (F_(1,30)_ = 186.7; p < 0.001). Moreover, the interaction of both variables was statistically significant (F_(4,30)_ = 32.2; p < 0.0001). Only moss discs were used for carbohydrate analysis. On average, for the five water levels, NSC were significantly lower in *S. uncinata* (20.9 ± 1.35 mg g^−1^ DW) than in *P. alpinum* (53.49 ± 1.35 mg g^−1^ DW) (Additional file 1: Table S1). During desiccation, NSC content decreased significantly by 50 % in *S. uncinata* from fully hydrated to desicccated (D100 to D0; Additional file 1: Table S1). By full rehydration (from R0 to R100), NSC values had increased significantly by 84 % (with the highest proportion as galactinol) (Fig. 2; Additional file 1: Table S1). In this moss, NSC content was similar in range (26–24 mg g^−1^ DW) for both the initial (D100) and the final state (R100).

Significant changes in NSC content were also found in *P. alpinum* (Additional file 1: Table S1) with a similar 54 % decrease of NSC level observed during desiccation from D100 to D0. In contrast to *S. uncinata*, during full rehydration (R0 to R100), NSC content increased only slightly reaching only 58 % of the pre-desiccation value (D100; Additional file 1: Table S1).

Galactinol was the most abundant NSC in both mosses, comprising about 27 % in *S. uncinata* during all stages of dessication and ca. 37 % during rehydration, without significant changes. In *P. alpinum* however, galactinol showed significant changes due to the treatment, with higher levels during desiccation (ca. 39 % of NSC), but decreasing concentrations during the rehydration process (19 %, Additional file 1: Table S1).

The composition of carbohydrates was similar in both moss species, including sugars of the sucrose (glucose and fructose) and raffinose (stachyose and verbascose) families (Additional file 1: Table S1). We also found a series of sugar alcohols (polyols: galactinol, adonitol, arabitol, and mannitol) (Additional file 1: Table S1). Only a few soluble carbohydrates (sucrose, verbascose, adonitol, arabitol) changed significantly in quantity between treatments in *S. uncinata* (Additional file 1: Table S1; P < 0.05, Fig. 2). In contrast, in *P. alpinum*, 11 of the 15 analyzed sugars showed significant differences (P < 0.001, Additional file 1: Table S1; Fig. 2). Three carbohydrates (pinitol, nystose, and kestose) were not detected (data not shown), and erythritol was only present in *P. alpinum*. In general, in both mosses the NSC content changed during desiccation and full rehydration. This effect was most siginificant when comparing starting and end point of treatments, whereas when mosses contained 50 % of water (at D50 and R50), NSC content was similar (Additional file 1: Table S1). Between the start and end of desiccation, carbohydrates varied in both species: with sucrose, fructose and glucose all declining significantly during desiccation in *P. alpinum*, while sucrose declined significantly in *S. uncinata* (Additional file 1: Table S1; Fig. 2). The disaccharide galactose increased notably more in *S. uncinata* than in *P. alpinum*, but only in the latter this change was significant (Additional file 1: Table S1; Fig. 2). Within the RFOs family (stachyose and verbascose) both diminished (Fig. 2) considerably in *S. uncinata* (ca. 27 and 35 %) but only slightly in *P. alpinum* (19 and 20 %, Fig. 2). Sugar alcohols (adonitol and arabitol) increased in *S. uncinata* and decreased in *P alpinum* during water loss (Additional file 1: Table S1). In both mosses mannitol decreased during desiccation.

The opposite was found when mosses rehydrated from R0 to R100 (Additional file 1: Table S1; Fig. 2). In *S. uncinata*, sucrose increased and concomitantly fructose and glucose were depleted (Fig. 2), along with galactose (Fig. 2). In this species during the same process, stachyose, verbascose and galactinol increased (Fig. 2). In contrast, in *P. alpinum* during rehydration, while sucrose and glucose increased, only fructose decreased (Fig. 2). At the same time, verbascose and stachyose increased, whereas galactinol decreased (Additional file 1: Table S1; Fig. 2); we also detected the production of erythritol and the increase of adonitol, mannitol and arabitol during this process (Fig. 2; Additional file 1: Table S1).

## Discussion

Mosses as poikilohydric organisms are constantly subject to changes in water tissue content, with internal water maintaining equilibrium with the surrounding environment. Our studied species showed obvious differences in their response to experimental water loss and uptake: *S. uncinata* discs lost water considerably more slowly than *P. alpinum* (Fig. 1b), but individual gametophytes of each species showed the opposite pattern, with *S uncinata* desiccating faster than *P. alpinum* (Fig. 1a). The shorter water retention time of individual *S. uncinata* gametophytes as compared to *P. alpinum* can be explained by differences in functional micromorphology: *S. uncinata* is an ectohydric moss that absorbs and loses water solely through its surface as it does not possess a cuticle [8], whereas *P. alpinum* is an endohydric moss that is characterized by its rudimentary conductive tissues, analogous to the xylem and phloem of higher plants, and a thin cuticle [27, 28]. These structural differences would determine the rate at which hydric equilibrium can be achieved between tissue and relative environmental humidity. As *P. alpinum* turf is quite open, individual plants of *P. alpinum* likely show a higher capacity for water retention than *S. uncinata* which grow in dense carpets. Similarly, when comparing bryophytes growing in situ on the subantarctic Signy Island, *S. uncinata* and other ectohydric mosses such as *Schistidium antarctici* (Card.) L. Savic. and Smirn, *Calliergon sarmentosum* (Wahlenb.) Kindb, and *Chorisodontium aciphyllum* (Hook. f. and Wilson) all showed shorter desiccation times than endohydric mosses such as *P. alpinum* and *Polytrichum alpestre* [29, 30].

In the field, gametophytes of *S. uncinata,* a pleurocarpic species, form a compact carpet that reduces the exposed surface, thereby partially limiting water loss [29, 31], as can be observed by its slower desiccation rate when samples where collected as discs, keeping the agreggated form intact (Fig. 1b). According to Robinson et al. [24], the dynamics of desiccation in the field vary substantially between carpets and cushions and it is probably due to its dense growth form that *S. uncinata* discs can retain water for a longer period than *P*. *alpinum*. The rate at which both mosses lose and recover hydric status is not only related to structural resistance to water loss, it also determines the time available for the synthesis of compounds necessary for greater desiccation tolerance. The contrasting response to desiccation between both species can be related to carbohydrate metabolism, which changes during the treatments.

In contrast to vascular plants, we found that in these Antarctic moss species, non-structural carbohydrate (NSC) content decreases during desiccation (D100 to D0; Additional file 1: Table S1). This finding is in line with the report of Smirnoff [20] that during desiccation of three moss species, *Dicranum majus, Polytrichum formosum*, and *Tortula ruraliformis*, soluble sugars do not play an osmotic role during short-term water loss, as has been observed to occur in vascular plants [18]. In vascular plants, accumulation of soluble sugar in response to desiccation is an important mechanism for the adquisition of drought tolerance. In contrast to other reports, in our mosses NSC content decreased to ca. 50 % in *S. uncinata* and 40 % in *P. alpinum* (Additional file 1: Table S1). Moreover, we found that under laboratory conditions, fructose was higher than glucose or sucrose in both species, even though the principal and responding sugar reported in mosses under field experiments is sucrose [20, 24, 32]. Another marked difference is the presence of high levels of galactinol. This sugar alcohol was found in both mosses, a novel finding for the studied species. Interestingly, galactinol has been been linked in vascular plants to tissue viability following desiccation [33] and to drought tolerance in the desiccation-tolerant *Sporobolus stapfianus* [34]. Galactinol in *S. uncinata* represents about 27 % of all NSC during desiccation, increasing to 37 % during rehydration (Additional file 1: Table S1). In contrast, in *P. alpinum*, galactinol represents an even bigger proportion (about 39 %) of NSC during desiccation but decreases to 19 % during the rehydration process. This suggests different functional roles of galactinol during desiccation and rehydration in both species: in ectohydric *S. uncinata*, galactinol should favor water uptake during rehydration having an osmotic function, whereas in endohydric *P. alpinum* galactinol probably acts as an osmoprotector avoiding damage of membranes during water loss, while during rehydration this sugar is not necessary at high level.

Our results suggest that the biosynthetic pathway of RFOs in the examined bryophytes is active [35, 36]. In *P. alpinum* and *S. uncinata*, the presence of raffinose was not detected, however, stachyose and verbascose were found. The absence of raffinose suggested that it was depleted to form other RFOs units, especially since field experiments in the Antarctica have shown the presence of both carbohydrates during long term in situ desiccation [40]. Stachyose has been previously reported in low concentrations in Antarctic mosses [24]. In two vascular, resurrection plants, *Boea hygroscopica* and *Haberlea rhodopensis,* levels of stachyose and verbascose became significantly elevated under severe desiccation stress [35, 41]. The response to desiccation was mediated by the interplay of several groups of carbohydrates in both species. The RFO, sugar group represents a high proportion of non-structural carbohydrates in both these moss species, playing an important role during desiccation (Additional file 1: Table S1; Fig. 2), with decreasing verbascose and stachyose level during water loss. In contrast, whereas verbascose and stachyose increased in *S. uncinata* during rehydration, in *P. alpinum* this was accompanied by an increase in galactinol. It has been reported, that RFOs sugars also accumulate during desiccation in seeds of various angiosperms [37] and that they are active in higher plants exposed to cold stress [38]. Moreover, in vascular plants they have been shown to be involved in protecting membrane integrity and in cryoprotection, in addition to playing an important role as reserve sugars at low temperatures when starch cannot be used [39]. The high values of RFOs in both mosses suggest, that during full hydration (D100), verbascose and stachyose accumulate as storage sugars which are used during the water loss process, probably helping to stabilize macromolecules together with polyols.

Polyols (galactinol, mannitol, adonitol, arabitol, erithritol) play an important role in desiccation tolerance, probably acting as compatible solutes in the stabilization of macromolecules [42, 43]. The presence of polyols such as adonitol, arabitol, and mannitol has been described for other liverworts and Antarctic mosses, including *Cephaloziella exiliflora, Bryum pseudotriquetrum*, and *Grimmia antarctici* [24, 32]. However, the current report is the first to relate these sugar alcohols with processes of desiccation or rehydration. Clearly, polyols act principally in *P. alpinum*, where mannitol and arabitol have been depleted during desiccation; in contrast during water uptake, all four polyols increased considerablely, suggesting an osmotic functioning (Additional file 1: Table S1).

During water stress, carbohydrates represent a source of energy for the cell and protection for molecules, thereby decreasing the effects of water loss. In contrast to higher plants where sugars retain water through the formation of hydrogen bonds, in mosses, sugar hydrogen bonds can act as substitutes of water molecules lost during desiccation, thus maintaining the native form and activity of proteins [18, 44]. In Antarctica, mosses are not only exposed to water stress, but also to low temperatures and daily freeze–thaw cycles that impose a strong pressure on metabolism, which must continuously adjust to avoid water loss and cell damage.

It is evident that the metabolism of sugars in bryophytes is much more complex than previously assumed, especially given that recent reports have found that other moss species, grown under different conditions, are able to synthesize a series of new compounds, some of which were not previously described and which would have distinct roles in metabolic processes [45]. This creates new questions for carbohydrate metabolism in Antarctic mosses exposed to cold, freezing, and drought conditions.

## Conclusions

*Sanionia uncinata* and *P. alpinum* presented differences in water loss and retention capacities. *S. uncinata* showed the strongest contrasting responses between plant form, with individual plants losing water rapidly while grouped discs were able to maintain a high water content over a longer period. Individual plants of *P. alpinum*, which have a rudimentary vascular system, were able to maintain water content longer than *S. uncinata*. Interestingly, both moss species showed insignificant changes in NSC contents after 50 % desiccation, only changing the level of carbohydrates during full water loss. The RFOs family of carbohydrates changed during desiccation and rehydration, and galactinol probably plays an important role during water management in both species. Differences in water loss and uptake can explain the different preferential growth sites for each moss, with *S. uncinata* growing in the flooded, sandy soil of valleys fed by run-off water from glaciers or snow banks and *P. alpinum* growing in small cushions dispersed on sandy soil without a continuous water supply. The high capacity of *S. uncinata* to maintain water for a longer time suggests that this moss species could play an important ecological role in the Antarctic tundra ecosystem, where it would provide other species with an additional water supply during drought periods; this could also partially explain the dominance of *S. uncinata* in large tundra communities of Fildes Peninsula on King George Island, as well as on other islands of the South Shetland Island Archipelago.

## Methods

### Plant samples

Gametophytes of *S. uncinata* (Hedw.) Loeske and *P. alpinum* (Hedw.) G.L. Smith were collected during summer 2013 at Juan Carlos Point (S62°12.03′ W058°59.66′) on Fildes Peninsula, King George Island in the South Shetland Islands Archipelago. The identification of each species was performed through microscopic analysis according to Ochyra et al. [8]. The samples were kept dry until used in desiccation and rehydration experiments. Reference specimens of each moss were deposited at the herbarium of the Universidad de Concepcion, CONC.

### Experimental design

We determined first the water loss time for a) individual gametophytes of plants and b) discs consisting of various gametophytes of both species. We took discs with a punch directly from carpets (agreggated form) of *S. uncinata* or from cushions of *P. alpinum*. Disc sample sizes where similar, of 10 mm diameter (area = 78.5 mm^2^), 10–15 mm in height, and 0.2–0.3 g of dry weight for *S. uncinata* and 0.30–0.35 g dry weight for *P. alpinum*. The mosses were rehydrated through submersion in distilled water for 30 h at 6–8 °C while being illuminated with photosynthetic active radiation of 100 μmol/m^2^ s^−1^, in order to promote an active metabolism prior to desiccation. Following this, the superficially accumulated water was removed using a paper towel. Both, discs (four replicates) and individual (6 replicates) gametophytes were submerged in distilled water, dried at room temperature, and weighed during the entire process with a model M2P analytical microbalance (SARTORIUS, Germany). Given the high rate of water loss in individual gametophytes and the high sensitivity of the microbalance, the entire process of dehydration was recorded uninterruptedly using a DSC-S730 video camera (Sony, Japan). Following this, the video was reviewed, and mass was recorded every 5 min. The hydric content was calculated based on decreasing mass in mg H_2_O g^−1^ DW.

For the second experiments we used only discs of gametophytes. For the dehydration and rehydration we established three levels (a) completely hydrated, 100 % H_2_O (D100); (b) moderately hydrated, 50 % H_2_O (D50); and (c) dry, 0 % H_2_O (D0). For this purpose, moss samples were dehydrated in a glass desiccator with desiccant agent silica gel and weighed using an analytical WTB 200 balance (RADWAG, Poland). After complete loss of water, the disc samples were rehydrated. For this, disc samples of both moss species were partially submerged in water so that hydration occurred through capillarity. The starting point of rehydration corresponded to the most desiccated treatment, (D0) but in the case of rehydration, this point was established as R0. Samples were rehydrated to reach 50 % (R50) and 100 % (R100) hydric content. For each treatment, four replicate individuals per species were used, and likewise, four tissue samples were collected for analysis of soluble sugars. Hydric content, expressed as  % of H_2_O, was determined by using dry weight (DW) and fresh weight (FW) of each sample according to the following formula: (FW-DW) × 100 %/(FW).

### Quantifying soluble sugars

During the second experiment, we took samples for carbohydrate analysis from discs of both moss species. The extraction and quantification of total soluble sugars was performed according to Zúñiga et al. [40]. Briefly, 0.100 g of FW was taken for each desiccation and rehydration treatment, and this sample was incubated at 4 °C in 1 mL of 80 % ethanol for 96 h (4 days). For high performance liquid chromatography (HPLC) analysis, aliquots of 480 μL were concentrated (Savant DNA SpeedVac, Minn., USA) and then resuspended in 0.1 mM of calcium-EDTA buffer before being filtered (0.45 μm). A volume of 20 μL per sample was injected into an Agilent 1100 series chromatograph equipped with a 300 mm × 6.5 mm Sugar-pak I column (Waters Corp., Mass., USA) at 75 °C and with an Agilent 1100 series refractive index detector at 55 °C. The isocratic elution program consisted in a mobile phase of 0.1 mM calcium-EDTA, with a flow of 0.35 ml min^−1^ and a pressure of 38 bars per 40 min. To identify soluble carbohydrates standards of glucose, fructose, galactose, galactinol, sucrose, raffinose, stachyose, verbascose, nystose, kestose, adonitol, arabitol, erythritol, mannitol, and pinitol were used (Sigma, USA).

### Statistical analysis

The time variation in responses to treatments (five water levels), sample type (individual plants or disc samples) and species (*S. uncinata* and *P. alpinum*) were analysed with ANOVA (p < 0.05; CI 95 %); as well as to compare the differences in carbohydrates level due to treatments and species. Thereafter, we separated the analysis of changes for each soluble carbohydrate by species, using one-way ANOVA. For a multiple comparison of measurements according to statistical differences, Tukey’s test (P < 0.05; CI 95 %) was applied. Statistical analyses were performed using the InfoStat software [46].

## Additional file

10.1186/s40659-015-0058-z Non structural carbohydrate content (mg g^−1^ DW) of two Antarctic moss species expossed to desiccation and rehydration treatment under controlled conditions.
