# Supplementary material for: Soluble carbohydrate content variation in Sanionia uncinata and Polytrichastrum alpinum, two Antarctic mosses with contrasting desiccation capacities
Source: Biol Res. 2016 Jan 28;49:6. doi: 10.1186/s40659-015-0058-z (PMC4731983; doi:10.1186/s40659-015-0058-z)
Supplement: Supplementary file 1 — 10.1186/s40659-015-0058-z Non structural carbohydrate content (mg g−1 DW) of two Antarctic moss species expossed to desiccation and rehydration treatment under controlled conditions. [file 40659_2015_58_MOESM1_ESM.docx]

**Table 1.**

Non structural carbohydrate content (mg * g ^-1^ DW) of two Antarctic moss species expossed to desiccation and rehydration treatment under controlled conditions.

|  | **Desiccation** | | | | | **Rehydration** | | | | | **P** |
| --- | --- | --- | --- | --- | --- | --- | --- | --- | --- | --- | --- |
|  | **D100** | **%** | **D50** | **%** | **D0 (R0)** | **%** | **R50** | **%** | **R100** | **%** |  |
| ***Sanionia uncinata*** | | |  |  |  |  |  |  |  |  |  |
| Sucrose | 2.4 ± 0.7 b | 9.3 | 1.8 ± 0.3 ab | 8.4 | 1.1 ± 0.6 a | 9.0 | 2.2 ± 0.4 b | 5.0 | 2.8 ± 0.5 b | 6.0 | 0.003 * |
| Glucose | 1.5 ± 0.1 a | 5.2 | 0.7 ± 0.6 a | 3.3 | 0.8 ± 0.4 a | 8.0 | 0.8 ± 0.3 a | 4.2 | 1.1 ± 0.3 a | 4.6 | 0.153 |
| Fructose | 4.3 ± 2.9 a | 15 | 3.7 ± 0.6 a | 18 | 1.9 ± 0.8 a | 16 | 2.0 ± 0.4 a | 10 | 1.6 ± 0.8 a | 7.3 | 0.066 |
| Galactose | 1.4 ± 1.2 a | 5.7 | 2.5 ± 1.2 a | 11 | 2.1 ± 1.6 a | 12 | 1.2 ± 0.9 a | 5.5 | 1.3 ± 0.6 a | 5.2 | 0.463 |
| Stachyose | 0.7 ± 0.4 a | 2.6 | 0.5 ± 0.2 a | 2.4 | 0.3 ± 0.2 a | 1.9 | 0.6 ± 0.1 a | 3.0 | 0.5 ± 0.1 a | 2.1 | 0.127 |
| Verbascose | 1.1± 0.6 b | 4.3 | 0.8 ± 0.3 ab | 3.5 | 0.3 ± 0.2 a | 2.8 | 0.8 ± 0.1 ab | 4.3 | 0.8 ± 0.2 ab | 3.4 | 0.032 * |
| Galactinol | 7.6 ± 3.3 a | 29 | 5.8 ± 1.8 a | 27 | 3.9 ± 2.6 a | 27 | 7.3 ± 2.2 a | 37 | 9.0 ± 1.6 a | 37 | 0.077 |
| Adonitol | 3.2 ± 0.4 b | 13 | 2.7 ± 0.7 ab | 13 | 1.7 ± 0.5 a | 14 | 2.7 ± 0.5 ab | 14 | 3.0 ± 0.1 b | 13 | 0.004 * |
| Mannitol | 3.3 ± 0.2 a | 13 | 2.5 ± 0.2 a | 12 | 1.5 ± 0.2 a | 11 | 1.9 ± 0.1 a | 8.6 | 2.5 ± 1.7 a | 9.3 | 0.138 |
| Arabitol | 1.5 ± 0.1 bc | 6.5 | 1.0 ± 0.1ab | 5.1 | 0.7 ± 0.2 a | 7.1 | 1.8 ± 0.1 c | 11 | 1.8 ± 0.4 c | 7.3 | 0.002 * |
| **Total NSC** | **26.2 ± 7.5 a** | **100** | **21.4 ± 3.9 ab** | **100** | **13.2 ± 6.7 b** | **100** | **19.5 ± 4.1 ab** | **100** | **24.4 ± 4.7 ab** | **100** | **0.0404*** |
|  |  |  |  |  |  |  |  |  |  |  |  |
| ***Polytrichastrum alpinum*** | | |  |  |  |  |  |  |  |  |  |
| Sucrose | 6.7 ± 0.8 c | 8.6 | 6.3 ± 0.6 c | 8.1 | 3.3 ± 0.5 a | 9.1 | 4.1 ± 0.4 ab | 14 | 5.6 ± 0.5 bc | 12 | 0.000 * |
| Glucose | 4.4 ± 0.8 ab | 4.5 | 5.1 ± 1.5 ab | 6.5 | 2.0 ± 0.3 a | 5.5 | 1.9 ± 0.3 a | 6.7 | 6.2 ± 2.0 b | 14 | 0.002 * |
| Fructose | 11.6 ± 1.0 b | 15 | 10.4 ± 0.9 b | 14 | 6.2 ± 0.7 a | 17 | 4.9 ± 1.2 a | 17 | 5.7 ± 1.8 a | 12 | 0.000 * |
| Galactose | 3.0 ± 0.9 a | 3.8 | 3.6 ± 3.4 a | 4.6 | 2.4 ± 1.5 a | 6.3 | 4.2 ± 2.4 a | 14 | 2.2 ± 1.4 a | 4.8 | 0.673 |
| Stachyose | 2.8 ± 0.4 c | 3.6 | 2.8 ± 0.3 c | 3.6 | 1.0 ± 0.2 a | 2.9 | 0.9 ± 0.2 a | 3.1 | 1.8 ± 0.3 b | 4.1 | 0.000 * |
| Verbascose | 5.0 ± 1.6 bc | 6.4 | 6.6 ± 0.5 c | 8.5 | 1.9 ± 0.3 a | 5.1 | 1.6 ± 0.5 a | 5.5 | 3.4 ± 0.6 ab | 7.7 | 0.000 * |
| Galactinol | 30.7 ± 3.7 c | 39 | 28.1 ± 1.9 c | 36 | 14.7 ± 2.3 b | 40 | 5.4 ± 0.8 a | 19 | 8.5 ± 1.6 ab | 19 | 0.000 * |
| Adonitol | 6.9 ± 0.7 b | 9.0 | 6.6 ± 0.9 b | 8.5 | 3.7 ± 0.3 a | 10 | 4.5 ± 0.6 a | 16 | 6.7 ± 0.7 b | 15 | 0.000 * |
| Erythritol | 0.0 ± 0.0 a | 0.0 | 0.0 ± 0.0 a | 0.0 | 0.2 ± 0.2 ab | 0.5 | 0.4± 0.1 b | 1.3 | 0.4 ± 0.3 b | 0.9 | 0.047* |
| Mannitol | 4.7 ± 0.3 b | 6.0 | 3.9 ± 0.5 b | 5.0 | 2.0 ± 0.3 a | 5.0 | 2.0 ± 0.2 a | 6.3 | 3.8 ± 0.6 b | 8.5 | 0.000 * |
| Arabitol | 4.7 ± 0.5 b | 5.7 | 4.0 ± 0.5 b | 5.2 | 0.0 ± 0.0 a | 0.0 | 0.0 ± 0.0 a | 0.0 | 1.3 ± 1.6 ab | 2.7 | 0.000 * |
| **Total NSC** | **78.4 ± 10.1 a** | **100** | **77.5 ± 4.9 a** | **100** | **36.9 ± 5.5 bc** | **100** | **28.9 ± 3.0 b** | **100** | **45.8 ± 6.8 c** | **100** | **0.0001*** |

Values are means (n=4) ± SD. * indicates statistically significant differences (p ≤ 0.001) between conditions of hydric content as determined by one-way analysis of variance (ANOVA). Means with the same letter within a row indicate the lack of significant differences as determined through a Tukey test (P < 0.05). D0, D50, D100, R0, R50 and R100 represent the percentage of tissue water content.
